# Report of the Committee on Medical Education

**Published:** 1850-12

**Authors:** 


### ECLECTIC DEPARTMENT,

### AND SPIRIT OF THE MEDICAL PERIODICAL PRESS.

---

*Report of the Committee on Medical Education. Submitted at the meeting of the Am. Med. Association, May,* 1850.

The Committee on Medical Education, respectfully submit the following REPORT :

The subject of Medical Education has been thoroughly discussed in the Association, the profession, and the journals during the last three years. Three formal reports have been laid before the Assoeiation, containing a full account of the general condition of medical education in this country and abroad, representing its defects, and recommending various measures of reform. The Committee feel, therefore, that the Association is in possession of all the facts essential to a correct comprehension of American medical schools, and that they may safely omit from the present report, many of the more special subjects which have occupied the attention of their predecessors.

The records of the discussion thus far embrace, as in most cases where practical subjects have been submitted to abstract investigation, a somewhat intricate intermingling of *facts* and *opinions*.

The *facts* include a statistical history of the schools, professors, students, and graduates; their situation, quantity, kind and means of instruction; preliminary and ultimate requirements, mode of examination, and other important particulars.

The *opinions* relate chiefly to conclusions thought to be deducible from the *facts.* So far as they have assumed a definite form, they assert, first in general terms, that the system of medical education in this country is defective. Second, that it is so because the number of schools is too great, their situation (in many cases) bad, their instructors too few, their students and graduates too many, the time devoted to instruction too short, the quantity too limited, the quality too superficial, the bestowal of honors too profuse, and the mode of bestowal too unrestricted; all this involving, inevitably, a depreciation of the profession, and an impairment of the dignity, honor, and usefulness of the healing art.

The position of the Association, in relation to this subject, has been uniformly one of forbearance and conciliation. It has recommended that certain customs of the schools should be altered and amended. These recommendations refer mainly to the preliminary education of pupils, the requirements exacted from candidates for graduation, extension of the term of study, the manner in which the test for professional honors shall be applied, the mode in which teachers shall be appointed, and some incidental advice as to particular subjects of study.

The schools have been disposed to meet the efforts of the Association in a spirit of cordial co-operation. Some of them have attempted to carry into practice its most important recommendations; and almost all have manifested a desire to contribute to the improvement of professional education. In no instance has any one of them, so far as the Committee can learn, assumed a hostile aspect towards the Association or its objects. Whatever reproaches may have been uttered against them for deficiency or delinquency, they have reason to feel proud that their relation to the Association has been, from the first, one of harmony and good will; that they have identified themselves with it, not to thwart or defeat its great designs, but to further their fulfilment; not merely for their own honor and their own interest, but for the advancement of science and the good of their fellow men.

The chief purposes of the Association in connection with the subject of medical education, have been—

1st. To elevate the character of preliminary preparation.

2d. To secure to the student, during the portion of his pupilage passed in the public schools, competent and complete instruction.

3d. To provide for the application of a severer test of qualification for admission into the profession.

The Committee propose, therefore, to confine themselves to a concise consideration of these three points.

1*st. Preliminary Education.*

Neither public nor private teachers of medicine can deny that many of their pupils come to them imperfectly and insufficiently prepared. Aside from the deficiencies which immature age almost necessarily implies, it cannot be gainsayed that a considerable number of medical students in this country enter upon their professional education with a limited and inadequate amount of proper preliminary training. The fact admitted, the question follows, Where is the fault, and where lies the remedy? A full solution of this question would impose upon the Committee a more difficult duty than they desire or design to fulfil. They only suggest that the blame and the remedy should be equally shared by the *profession*, the *schools*, and the *student.*

The *profession*, occupying an intermediate relation to the student and the schools, should abstain from offering to the student any enticement or encouragement not based upon a right knowledge of his character and capacity.

The *schools* should have the firmness to reject all importunity not sustained by real and appreciable qualification. With reasonable allowance for difference of original capacity, means, and opportunity, it should be their habit, as it is their duty and their interest, to see that the reputation and usefulness of the profession, of which they are, in some sense, the recognized guardians, receive no detriment through the admission of the incompetent and unworthy.

The *student* should be told, frankly and plainly, by those who have the best right and opportunity to do it, that nothing can compensate for lack of physical, intellectual, and moral fitness for his vocation; that his position in a profession for which he has none of the essential pre-requisites, must ever be a false one, and that he has no just claim to its "honors and immunities," without faithful, continued, industrious application. The burden is a common one, and should be equally borne. So far as the schools are concerned, the best boon that could be bestowed upon them, would be to ensure to them intelligent, tractable, and ambitious pupils. No public teacher would reject such a gift. His work is attractive or repulsive, in exact proportion to the degree of mental correspondence between himself and those who listen to his instructions. To suppose that the schools do not desire the greatest amount of ability and culture in those who seek their halls, is as absurd as to suppose that the recruiting officer would prefer to enlist dwarfs, cripples, and idiots. There is not a teacher of medicine, from the St. John to the Sacramento, who would not confess that all the irksomeness of his daily duties, and all the doubts of his final decisions, are dependent upon the intrinsic and essential unfitness of the ignorant and the indolent; and that all the pleasures of a position which, however eagerly the ambitious aspirant may covet it, often involves much self-sacrifice, spring from his relations to the well-trained, the industrious, and the intelligent.

The Association has attempted to obviate this difficulty by urging upon the profession and the schools certain recommendations, which as yet have not been fully complied with. Neither of the parties most interested seems willing to assume the responsibility. The student dislikes to have his self-complacency offended; the practitioner fears to hazard the good will of the student; and the school, it is feared, is often too anxious, lest the portal of some active rival should be found of easier access than its own. Hence, with the defect generally admitted and deplored, it does not appear that hitherto much has been done towards applying any efficient remedy.

[Dr. Blatchford, of Troy, N. Y., a member of the Committee, states in a note appended to this report, that " local medical societies, in States where an organization has been effected, have, however, to a limited extent, adopted the plan of a preliminary board of examiners, a certificate from which is required of the pupil before entering the office of any one of the members as a student."]

2*d*. *The Association has endeavored to secure to the student competent and sufficient instruction, during that portion of his pupilage which is passed in the schools.*

The charge has been somewhat pertinaciously repeated that the student, after entering the schools, is often imperfectly and insufficiently taught; that there are schools badly situated, scantily officered, and meagrely equipped—unable, in short, to fulfil their promise to provide a proper course of public instruction. The Committee are not in possession of the name of any particular school against which, justly or unjustly, this charge has been openly and directly alleged. If there be a single school fairly deserving such an imputation, it is hoped that no false compassion will interpose to shield it. After three years of consultation and discussion, it surely cannot be deemed unreasonable that this Association should discriminate between those schools which have its confidence and those which have not.

Practically, the Association has advised an increase in the number of instructors, and an extension of the period of instruction. It is not known that either of these recommendations has been generally adopted; as to the last, it would seem that the original design of the Association, which, as the Committee apprehend, contemplated a lengthening of the term of lectures without materially increasing their number (on the ground that too many were crowded into too short a time,) has been somewhat misunderstood.

Since, of late, it has been argued that the *completeness* of a course of lectures necessarily depends upon its duration, and that this is an indisputable advantage of the six over the four months' course. The schools also appear to differ in their understanding of this point ; some of them having increased both the term and amount of instruction, while others have extended the former alone. Thus, in the institution selected by the Committee of 1849 "as a model of the schools of our own country, whose rules and requirements are believed to be quite as full and extensive as those of any other institution, and whose trustees and professors seem to be as well disposed as any others to accede to the general wish of the profession, as expressed through this Association, to favor proper measures of reform"—although the lecture term is one of six months, the aggregate amount of instruction, as reported, is really less than in another school whose course continues but four and a half months. The Committee would suggest, however, that it should be fairly stated, by those schools in which the session is prolonged, whether there is, or is not, a proportional increase of the amount of instruction.

*3d. The Association has tried to provide for the application of a more rigid test of qualification for admission into the profession.*

It has proposed that the profession itself should have a voice and a vote in deciding upon the worthiness, or unworthiness, of those who desire to enter it; and that, for this purpose, there should be boards of examiners, composed of teachers and practitioners. Aside from any inclination or disinclination in the schools to consent to this arrangement, one thing must be yielded, that in this country admission to all the liberal professions must always remain comparatively easy. The nature of all our institutions supposes this. They must conciliate the public good will, upon which they are dependent for existence and patronage, by a liberal exercise of their powers and privileges. This is especially the case with new institutions in new States. It may be deemed doubtful, therefore, whether any uniform plan of medical education, or any uniform method of regulating admission to the profession, can be established throughout the Union. The Northern and Middle States, having a dense and wealthy population, accustomed to educational institutions, and able as well as willing to sustain them, are in a very different condition from that of the newer communities of the west and south-west. To insist that regardless of these differences, all shall conform to an arbitrary standard, would be to insist, in the judgment of the Committee upon something utterly impracticable. A disparaging comparison has been drawn between the system of medical education in other countries and in this. Admitting that American and European medical schools differ in the amount of exact training, rigid requirements and restraining discipline considered essential to the highest grade of intellectual education, it does not follow that it is practicable or desirable to assimilate them in all respects. There is no central power in this country able to originate or enforce a uniform system of laws regulating matters pertaining to general or professional education; and it is probable that an attempt to establish such a system would at once excite suspicion and opposition. Indeed, it is notorious that the friends of the only institution in the country* that approaches the best foreign schools in thoroughness of scientific and

* The United States Military Academy.

practical instruction, have been repeatedly called upon to repel the assaults of political jealousy.

The Committee have no wish to excite profitless discussion. They do not deny that American medical education may fall short when compared with that of the best European schools. Yet, after all the reproaches that have been uttered, and all the delinquencies that have been alleged, it does not appear that more severely disparaging epithets have been applied, rightly or wrongly, to an American degree, than were so strongly bestowed upon certain foreign degrees some twenty years ago; or that, even now, there is more content in the profession in Great Britain, with its Royal Colleges of Physicians and Surgeons, or in France, Austria, and Prussia, with their stern restrictions as to what shall or shall not be done, and who shall and who shall not do it, than at present exists in the United States.

The Committee have forborne from a more elaborate examination of the subject entrusted to them, from a conviction that it would be impolitic to multiply issues upon which the profession and the schools may differ. As to present action, they would recommend that the Association await the result of its advice to the "various schools of medicine to meet at Cincinnati before the next (this) annual meeting, and recommend and present a plan for elevating the standard of medical education to this Association."

Having fully and freely reiterated its opinions and wishes upon the subject, the Association has a right to expect from the schools an equally frank expression of theirs.

By a resolution passed at the last annual meeting, this Committee were directed to inquire into "the expediency of establishing a school, or schools, of pharmacy in the respective States, for the special purpose of preparing persons for the business of apothecaries; and also the expediency of adopting a rule that no physician ought to patronize a druggist or apothecary who deals in secret or patent remedies."

Schools of pharmacy already exist in several of the Atlantic cities. In New York and Philadelphia, these institutions have acquired a deserved reputation for capacity, industry, and usefulness; and there can be no doubt that the organization of similar schools throughout the country would be productive of great benefit. At present, the occupation of "apothecary" is open to all who choose to follow it; and there is scarcely a town or a village, from Eastport to San Francisco, in which one cannot buy drugs of all varieties of quality, and poisons of all degrees of intensity, from persons of all grades of ignorance and knowledge.

The expediency of adopting a rule that "no physician ought to patronize a druggist or apothecary who deals in patent or secret remedies," depends, in the opinion of the Committee, entirely upon the probability of its general observance. If, in the judgment of the Association, the sale of a single pernicious nostrum is likely to be checked by such a rule, a true regard for the interest of the public and the profession obviously prompts its immediate enactment, and its enforcement by all the pains and penalties the Association may have the power to inflict.

JOSEPH ROBY, *Chairman.*

NOTE.—The author of this report deems it proper to make the following explanation.

As chairman of the Committee, he prepared the report, and, in consequence of his inability to attend the meeting, transmitted it to Dr. Drake, of Cincinnati, with a request that it might be laid before the Committee before it was presented to the Association. The names and residences of the whole Committee were stated in the communication to Dr. Drake, and the report was sent to him as Chairman of the Committee of Arrangements, he having given notice publicly that it was his duty to receive the reports of committees whose members might not be present.

It was the intention of the writer to leave the whole subject in the hands of the Association. The report of 1849 had almost completely exhausted all information in relation to the condition of medical education abroad; the statistics of American medical schools; the requirements of the army and navy boards; and the laws regulating the practice of medicine in the several states. It was not thought needful or desirable to go over this ground anew. Regarding the powers of the Committee as discretionary, it was hoped that the Association would not consider an omission of matters, which had been so fully treated of in previous reports, disrespectful to itself or a dereliction of duty in the Committee.

In the report, as read to the Association, an unintentional error existed which has been corrected. There was no motive or wish to do wilful injustice; the meaning of the writer was that but a limited number of the colleges of pharmacy "seem to be in active operation." In the transcription, the word "none" was unwittingly substituted for "few." The original opinion with regard to these institutions has since been fortified by the fact that, at the fourth decennial convention for reviewing the Pharmacopœia of the United States, which met at Washington on the 6th of May, 1850, but three of the whole number of schools and colleges of pharmacy in the country were represented; viz., the New York, Philadelphia, and Cincinnati Colleges.

